# Prospective assessment of serum neurofilament light chain in platinum-induced and taxane-induced peripheral neuropathy

**DOI:** 10.1136/bmjno-2025-001312

**Published:** 2026-01-05

**Authors:** Duncan Smyth, Ryan Y S Keh, Stephen Keddie, Michael Chou, Melanie Hart, Miles Chapman, Martin D Forster, Aisling Carr, Michael P Lunn

**Affiliations:** 1 Neuromuscular Diseases, UCL, London, UK; 2 Queen Square Centre for Neuromuscular Diseases, London, UK; 3 NHS Neuroimmunology and CSF Laboratory, UCL Queen Square Institute of Neurology, London, UK; 4 Neuroinflammation, UCL, London, UK; 5 UCL Cancer Institute, London, UK; 6 Medical Oncology, University College London Hospitals NHS Foundation Trust, London, UK

**Keywords:** NEUROPATHY, NEUROTOXICOLOGY, NEUROONCOLOGY, ONCOLOGY

## Abstract

**Introduction:**

Chemotherapy-induced peripheral neuropathy (CIPN) is a potentially disabling complication of the neurotoxic chemotherapies; however, its occurrence is often unpredictable. We aimed to determine whether serum levels of neurofilament light chain (sNfL) could predict the onset and severity of CIPN, and whether sNfL levels were associated with other clinical factors in people with cancer.

**Methods:**

Adult patients (>18 years) prescribed at least four cycles of oxaliplatin, cisplatin, docetaxel or paclitaxel were clinically assessed and had blood taken for sNfL analysis at baseline and prior to each cycle. Peak sNfL was compared with clinical characteristics and Total Neuropathy Score-Clinical version (TNSc), National Cancer Institute Common Terminology Criteria for Adverse Events (CTCAE, V.4.03) and CIPN-Rasch-built Overall Disability Scale (CIPN-RODS). Individual patient trends in sNfL and TNSc were examined.

**Results:**

42 patients completed the study, with 36 receiving platinum agents and 6 receiving taxanes. Peak sNfL was higher with taxanes than with platinum agents (129.9 vs 31.0 pg/mL; p<0.0001). Higher peak sNfL was not associated with final TNSc, CTCAE or CIPN-RODS in patients receiving platinum agents. Using age-adjusted NfL z-scores, peak sNfL was associated with CIPN-RODS (rs=−0.45; p=0.012) and was higher in patients with a final CTCAE Grade of 2 compared with Grades 0–1 (p=0.015) but was not associated with final TNSc (rs=+0.37, p=0.050). In patients receiving platinum agents, higher peak sNfL was associated with death within 6 months of study entry (p=0.020). sNfL rose in conjunction with the increase in TNSc but did not precede clinical symptoms/signs of neuropathy in most patients.

**Conclusion:**

Taxanes cause greater and sharper sNfL rises than platinum agents. Age-adjusted sNfL associates with neuropathy severity in platinum-treated patients; however, in most patients it is unable to detect early axonal damage before this is detectable with clinical examination.

**WHAT IS ALREADY KNOWN ON THIS TOPIC**

- Serum neurofilament light chain (sNfL) is associated with chemotherapy-induced peripheral neuropathy (CIPN); however, its overall utility in the clinical setting assessing individuals has not been fully evaluated.

**WHAT THIS STUDY ADDS**

- Axonal damage is likely greater with taxanes than with platinum agents, suggesting that sNfL is likely to be more useful for taxane-associated than platinum-associated neuropathy. In most patients, serum NfL rises alongside clinical features of CIPN, but not before.

**HOW THIS STUDY MIGHT AFFECT RESEARCH, PRACTICE OR POLICY**

- Future studies should further examine the associations between NfL and adverse features of cancer in larger cohorts of CIPN.

## Introduction

 Chemotherapy-induced peripheral neuropathy (CIPN) is a common unwanted effect of neurotoxic chemotherapies, occurring in around 30–40% of those treated.^1^ CIPN is often mild and improves with time but can sometimes be severe and permanent, leading to chronic sensory loss, pain, weakness and impairment of function.^2^ In the majority of cases, the occurrence and severity of CIPN are unpredictable.^3^ A reliable, sensitive and specific serological biomarker of neuronal damage, preferably one that could predict the development or the potential severity of the neuropathy, would therefore be worthwhile. The development of a neuropathy often leads to chemotherapy dose reductions or cessation, which can impact survival from the malignancy,^4 5^ and thus quantifying mild or more severe damage which might guide chemotherapy reduction could be useful.

Neurofilament light chain (NfL) is a neuronal cytoskeletal protein that is released into interstitial fluid following axonal damage.^6^ It is the most well-known neurological biomarker, and its levels rise in the cerebrospinal fluid and serum in over 80 diseases of the central^6^ and peripheral nervous system.^7^

A number of studies have examined NfL in CIPN with some promising results. A study of 43 patients treated with 6 months of oxaliplatin for colorectal cancer found that serum NfL (sNfL) levels were higher in patients with National Cancer Institute Common Terminology Criteria for Adverse Events (NCI-CTCAE) Grade 3 peripheral neuropathy compared with Grades 0–2 after 6 months.^8^ Studies of paclitaxel treatment have found that sNfL levels increase as chemotherapy continues, often rising early in the chemotherapy course, with higher levels correlating with severity of neuropathy.^9^,^16^ sNfL has also been found to be associated with the CIPN caused by bortezomib.^17^

Although NfL can now be measured easily in the serum or plasma, and high NfL levels are associated with many different neuropathic conditions,^7^ it has not so far entered routine clinical practice for any form of neuropathy and its correlation with symptom onset and severity has not been clearly delineated. A limitation of NfL is that it cannot differentiate between central and peripheral nervous system axonal damage, and thus is difficult to interpret in the presence of other known or unknown neurological disorders. Levels are also known to increase with age.^7^ It is uncertain whether NfL can be used to predict the severity of different peripheral neuropathies or monitor patients on treatment in a sensitive and clinically informative way.^7^ In one study of CIPN, a sNfL of >85 pg/mL after 3 weeks of weekly paclitaxel provided only a low sensitivity (46.2%) to predict severe neuropathy at the end of treatment.^9^ Another study of paclitaxel used a cut-off of 73.4 pg/mL prior to cycle 2 and found a relatively low positive predictive value (68%) and negative predictive value (66%) to predict Grade 2–3 CIPN at the end of treatment.^14^ Thus, while NfL shows some promise as a biomarker for CIPN, it is still uncertain whether it will become a clinically useful predictive biomarker in the oncology clinic. Additionally, NfL levels may be raised by systemic insults and have been shown to be elevated in people with cancer postoperatively compared with preoperatively.^10 11^ How comorbidities in people with cancer influence NfL levels and relate to the development of neuropathy has not been systematically investigated.

This study aimed to determine: (1) whether serum levels of NfL correlate with clinical measures of neuropathy in patients receiving platinum or taxane agents, (2) if sNfL is predictive and might identify early axonal damage prior to the onset of clinical neuropathic symptoms and signs and (3) whether clinical factors other than neuropathy in people receiving chemotherapy are associated with sNfL levels.

## Methods

### Study participants

Adult patients (>18 years) were prospectively recruited from gastrointestinal, lung, head and neck and urological oncology clinics at University College London Hospitals NHS Foundation Trust, London, UK between April and October 2021. Patients were eligible for inclusion in the study if they were prescribed chemotherapy with oxaliplatin, cisplatin, docetaxel or paclitaxel with a minimum of four cycles planned. There were no specific exclusion criteria, and patients who had received previous chemotherapy or who had other causes for neuropathy (eg, diabetes mellitus) were eligible for inclusion.

### Data collection and clinical assessment

All clinical assessments were performed by a consultant peripheral nerve neurologist (DS). Data were collected on age, sex, ethnicity, body surface area (BSA), cancer type, cancer stage (localised vs metastatic), treatment intent (curative vs palliative), previous neurotoxic chemotherapy, diabetes mellitus, chronic kidney disease (CKD), neurotoxic drugs and alcohol intake. These data were collected from patient interviews and hospital medical records. At baseline, patients underwent a full neurological examination to determine if there was any neurological deficit and its anatomical localisation. In addition, the Total Neuropathy Score-Clinical version (TNSc),^18^ CIPN-Rasch-built Overall Disability Scale (CIPN-RODS)^19^ and CTCAE V.4.03 for motor and sensory peripheral neuropathy^20^ were assessed as neuropathy outcome measures. The TNSc is scored 0–28 and incorporates symptoms (sensory, motor and autonomic) and examination signs (pinprick, vibration, muscle strength, reflexes), with higher scores reflecting more severe neuropathy. The CIPN-RODS is a patient-reported outcome measure designed to assess function and disability from CIPN (scored 0–56), with lower scores reflecting greater disability. The CTCAE grades sensory and motor neuropathy severity from 0 (no evidence of neuropathy) to 5 (death).

Prior to subsequent cycles of chemotherapy, patients completed a brief 10-point screening questionnaire which contained relevant questions modified from the Michigan Neuropathy Screening Instrument,^21^ to screen for symptoms suggestive of neuropathy (online supplemental table 1). If the questionnaire identified any symptoms of neuropathy, a TNSc and CIPN-RODS were performed before that cycle and all subsequent cycles. If the patient had no symptoms suggestive of neuropathy and had had no symptoms during all previous cycles, they were assumed not to have significant neuropathy and did not undergo further assessment until the screening questionnaire was repeated before the following cycle. By definition, those without any symptoms had a CTCAE Grade of 0 or 1. CTCAE Grades of 0 and 1 were thus grouped for the analysis on the basis that significant neuropathy symptoms occur with a Grade of 2 or above. Patients who had their chemotherapy stopped early also underwent an assessment after their final cycle, timed to coincide with another hospital appointment, which was usually several weeks after the chemotherapy dose. Death within 6 months of study entry was recorded from hospital medical records. Most patients receiving oxaliplatin experienced transient sensory symptoms for several days following each cycle due to acute peripheral nerve hyperexcitability^22^—these were not scored as sensory symptoms for the purposes of calculating the TNSc unless they were persistent.

### Sample collection and NfL measurement

Patients had blood taken at baseline and before every subsequent chemotherapy cycle. Samples were usually obtained at the same time as standard prechemotherapy blood tests. However, if patients forgot to bring their research blood request form to the phlebotomy appointment, we took the sample on the day of chemotherapy, always prior to infusion of any drugs. Those who had chemotherapy stopped early also had blood taken after their final dose, usually several weeks postdose and timed to coincide with another hospital appointment. Serum samples were centrifuged, stored at −80°C then tested for NfL using the Simoa Nf-light kit on a Simoa HD-X analyser (Quanterix, Boston, Massachusetts, USA) after all clinical assessments had been completed.

### Statistical analysis

GraphPad Prism V.10.6.1 (San Diego, California, USA) was used for analysis. Normality was assessed using the Shapiro-Wilk test. The results are presented as mean±SD or median (IQR). We calculated that a sample size of 88 was required to detect a difference between CTCAE Grade 2 neuropathy and lower grades with a power of 80%, assuming an alpha of 0.05. Patients who received platinum drugs and patients who received taxanes were analysed separately due to the different mechanisms of these agents. Clinical characteristics were compared between platinum and taxane groups using Fisher’s exact test or Mann-Whitney U as appropriate. The Wilcoxon matched pairs signed rank test was used to compare sNfL concentrations at baseline with concentrations at later cycles. CTCAE was separated into two groups: 0–1 (no or non-significant neuropathy) and ≥2 (significant neuropathy), and TNSc was separated into severity quartiles as has been described by others: I (scores 1–7), II (8–14), III (15–21) and IV (>21).^9^

Peak sNfL was used for the analysis as it would most likely reflect the maximum extent of axonal damage. Patients who had no symptoms for their entire chemotherapy course (ie, all screening questionnaires were negative) did not have TNSc or CIPN-RODS done after baseline and thus were not included in comparisons of TNSc and CIPN-RODS. For the primary analysis, unadjusted peak sNfL values were compared with clinical characteristics and neuropathy outcome measures taken at the final patient assessment. Group comparisons were assessed using Mann-Whitney U and Kruskal-Wallis, and associations with continuous variables were assessed using Spearman’s correlation. For the secondary analysis, age-adjusted sNfL z-scores were generated (https://shiny.dkfbasel.ch/baselnflreference/)^23^ and compared with the clinical characteristics and neuropathy outcome measures. Group comparisons for NfL z-scores were assessed using Welch’s unpaired t-test and one-way analysis of variance as sNfL z-scores are adjusted to a normal distribution, and associations with continuous variables were assessed using Spearman’s correlation.

Individual patient trends in TNSc and sNfL were examined for both platinum and taxane-treated patients to determine whether increases in sNfL occurred prior to increases in TNSc, and thus whether sNfL could identify early axonal damage before it became clinically apparent.

## Results

### Patients and clinical characteristics

**Figure 1:**
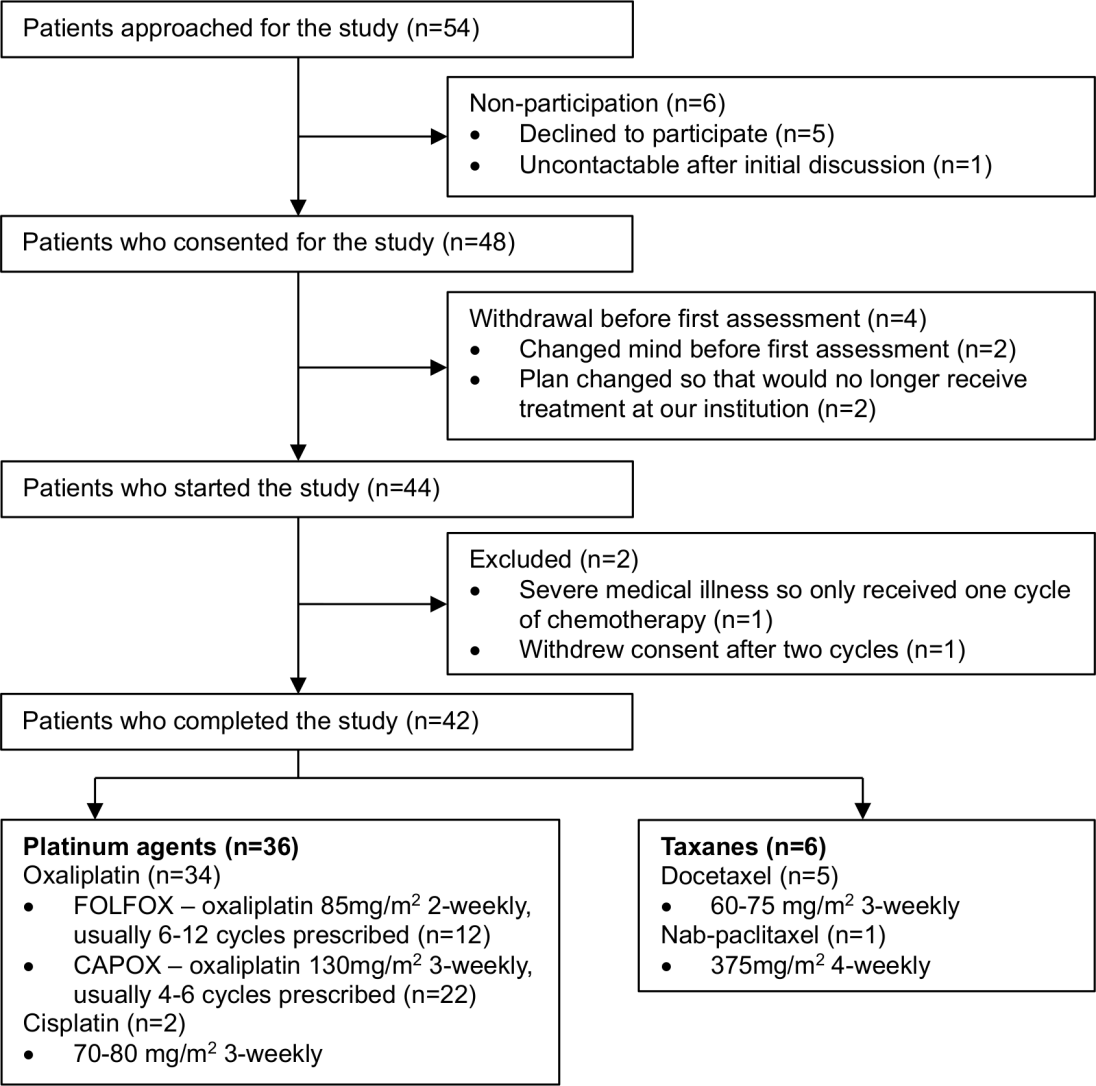
Flow diagram of patients included in the study and details of chemotherapy regimens. CAPOX, capecitabine/oxaliplatin; FOLFOX, folinic acid/5-fluorouracil/oxaliplatin.

42 patients completed the study; reasons for non-participation and withdrawal are detailed in figure 1. Of 162 assessments where TNSc was determined, 6 (3.7%) could not be done in person. In these cases, the assessment was done over the telephone and the examination findings were imputed as the last measurement brought forward. The majority of patients (86%) received platinum agents, mostly oxaliplatin (81%). Due to low numbers of patients receiving taxanes (n=6, 14%), the analysis primarily focused on platinum agents. Details of the chemotherapy regimens are contained in figure 1. 17 patients (40%) had chemotherapy stopped early and a further 6 (14%) had dose reductions due to various factors including reported neuropathic symptoms, other adverse effects, disease progression or death. Patients receiving taxanes were older compared with those receiving platinum agents (74.7 vs 60.9 years; p=0.0005), were more likely to have metastases (p=0.032) and be receiving chemotherapy with palliative intent (p=0.007), and had a higher median baseline TNSc (4.5 vs 1; p=0.046). We considered two patients (who both received platinum agents) to have symptomatic neuropathy at baseline, evidenced by both symptoms and signs of peripheral neuropathy. Five of the six patients with diabetes had minor examination abnormalities at baseline suggesting early neuropathy; however, none had any symptoms to suggest a clinically important neuropathy. Patient characteristics are summarised in table 1.

**Table 1:** Patient characteristics

Characteristic	Platinum agents (n=36)	Taxanes (n=6)	Total (N=42)	P value
Age, mean±SD (range)	60.9±11.0 (30–77)	74.7±5.4 (64–79)	62.8±11.4 (30–79)	0.0005
Sex (% male)	Male 21 (58)	Male 5 (83)	Male 26 (62)	0.38
	Female 15 (42)	Female 1 (17)	Female 16 (38)	
Body surface area, m^2^, mean±SD (range)	1.86±0.20 (1.47–2.25)	1.86±0.15 (1.62–2.01)	1.86±0.20 (1.47–2.25)	0.76
Cancer type	Colorectal 30 (83%)	Prostate 4 (67%)	Colorectal 30 (71%)	N/A
	Upper GI 3 (8%)	Upper GI 1 (17%)	Upper GI 4 (10%)	
	Bladder 2 (6%)	Lung 1 (17%)	Prostate 4 (10%)	
	Head and neck 1 (3%)		Other 4 (10%)	
Neurotoxic drug	Oxaliplatin 34 (94%)	Docetaxel 5 (83%)	Oxaliplatin 34 (81%)	N/A
	Cisplatin 2 (6%)	Paclitaxel 1 (17%)	Docetaxel 5 (12%)	
			Cisplatin 2 (5%)	
			Paclitaxel 1 (2%)	
Cancer stage	Localised 24 (67%)	Localised 1 (17%)	Localised 25 (60%)	0.032
	Metastatic 12 (33%)	Metastatic 5 (83%)	Metastatic 17 (40%)	
Treatment intent	Curative 28 (78%)	Curative 1 (17%)	Curative 29 (69%)	0.007
	Palliative 8 (22%)	Palliative 5 (83%)	Palliative 13 (31%)	
Number of cycles, median (IQR, range)	4 (4–5.75, 2–12)	4.5 (3–6.25, 3–7)	4 (4–6, 2–12)	0.92
Months of treatment, mean±SD (range)	3.0±0.96 (1.5–6)	3.6±1.1 (2.25–5.25)	3.1±0.99 (1.5–6)	0.19
Total dose, mg/m^2^, mean±SD (range)	Oxaliplatin 505.1±162.4 (238–1020)	Docetaxel 334.5±105.7 (217.5–435)	N/A	N/A
	Cisplatin 285	Paclitaxel 1125		
Cumulative dose at final assessment, mg/m2, mean±SD (range)	Oxaliplatin 429.6±153.6 (170-935)	Docetaxel 300±90.6 (180-375)	N/A	N/A
	Cisplatin 250	Paclitaxel 750		
Neuropathy risk factors	DM	DM	DM	0.57
	Yes 6 (17%)	Yes 0 (0%)	Yes 6 (14%)	
	No 30 (83%)	No 6 (100%)	No 36 (86%)	
	Prior chemotherapy	Prior chemotherapy	Prior chemotherapy	0.56
	Yes 4 (11%)	Yes 1 (17%)	Yes 5 (12%)	
	No 32 (89%)	No 5 (83%)	No 37 (88%)	
	Alcohol >30 units/week	Alcohol >30 units/week	Alcohol >30 units/week	>0.99
	Yes 4 (11%)	Yes 0 (0%)	Yes 4 (10%)	
	No 32 (89%)	No 6 (100%)	No 38 (90%)	
	CKD	CKD	CKD	>0.99
	Yes 2 (6%)	Yes 0 (0%)	Yes 2 (5%)	
	No 34 (94%)	No 6 (100%)	No 40 (95%)	
Death within 6 months	Yes 6 (17%)	Yes 1 (17%)	Yes 7 (17%)	>0.99
	No 30 (83%)	No 5 (83%)	No 35 (83%)	
Baseline TNSc (IQR, range)	1 (0.25–3, 0–9)	4.5 (1.5–7, 0–7)	1.5 (0.75–3, 0–9)	0.046

Continuous data are reported as means±SD, discrete data are reported as medians (IQR).

CKD, chronic kidney disease; DM, diabetes mellitus; GI, gastrointestinal; N/A, not assessed; TNSc, Total Neuropathy Score-Clinical version.

### sNfL changes with time

**Figure 2:**
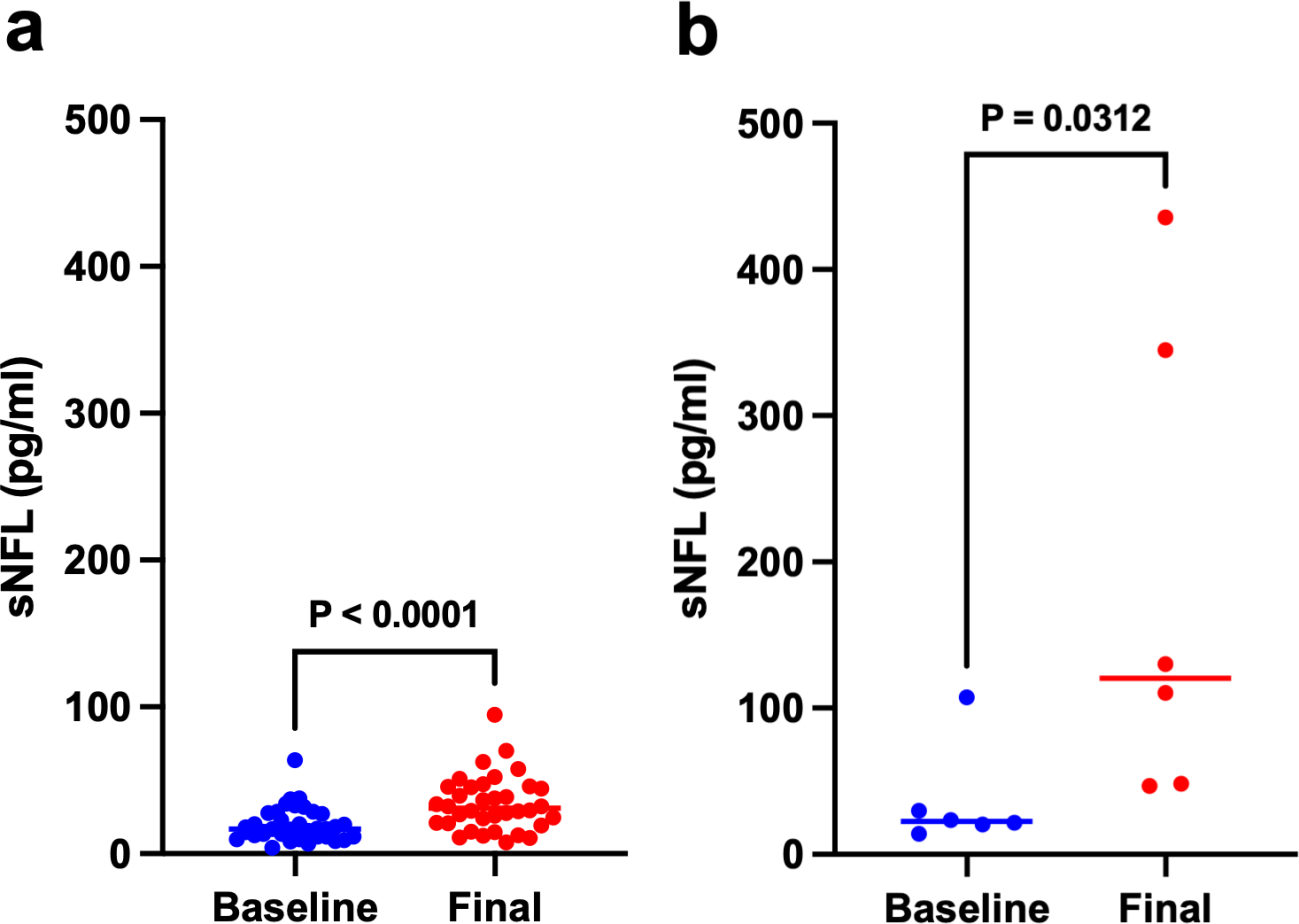
Median sNfL increased from 16.8 pg/mL (pretreatment) to 31.0 pg/mL (final measurement) in patients receiving platinum agents (**a**). In patients receiving taxanes, median sNfL increased from 22.5 pg/mL (pretreatment) to 120.3 pg/mL (final measurement). Final sNfL was higher in patients receiving taxanes than in patients receiving platinum agents (p=0.0001) (**b**). sNfL, serum neurofilament light chain.

In patients who received platinum agents, the median baseline sNfL concentration was 16.8 pg/mL (IQR 11.6–27.5, range 4.2–63.9), the final sNfL concentration was 31.0 pg/mL (IQR 20.9–45.4, range 7.8–94.5; p<0.0001) and the peak sNfL concentration was 31.0 pg/mL (IQR 21.1–47.7, range 8.2–94.5; p<0.0001). In patients who received taxanes, the median baseline sNfL of 22.5 pg/mL (IQR 18.9–49.3, range 14.2–107.3) was similar to those who received platinum agents (p=0.095); however, final (120.3 pg/mL, IQR 47.9–367.5, range 46.7–435.6; p=0.0001) and peak sNfL (129.9 pg/mL, IQR 65.1–436.9, range 48.3–440.7; p<0.0001) were significantly higher (figure 2). For contextual reference, in the normal 60–69 years old population, sNfL is 3.97–22.27 pg/mL.^24^ Findings were consistent when using sNfL z-scores in place of unadjusted sNfL values.

**Figure 3:**
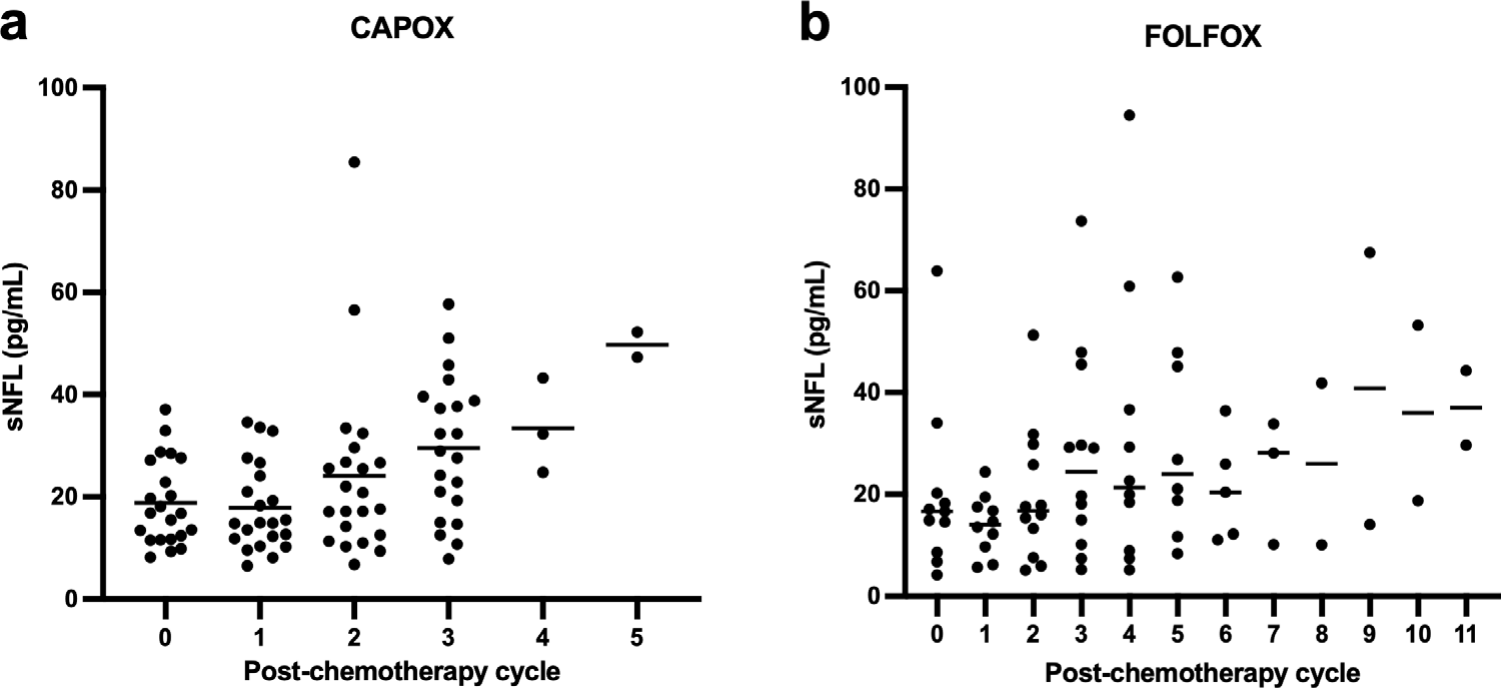
Serial sNfL concentrations in patients receiving oxaliplatin (n=34). In patients who received CAPOX (capecitabine/oxaliplatin), sNfL was significantly higher after three cycles (median 28.9 pg/mL, IQR 17.1–39.2, range 7.84–57.7, n=21) compared with baseline (median 16.8 pg/mL, IQR 11.7–27.3, range 8.18–37.1; p<0.0001) (**a**). In patients who received FOLFOX (folinic acid/5-fluorouracil/oxaliplatin), sNfL was significantly higher after five cycles (median 24.0 pg/mL, IQR 13.5–47.1, range 8.40–62.7, n=8) compared with baseline (median 16.6 pg/mL, IQR 8.64–20.2, range 4.17–63.9; p=0.016) (**b**). sNfL, serum neurofilament light chain.

The mean number of blood samples taken per patient was 4.9 (range 3–11). Out of 217 possible samples, 207 (95%) were taken and tested—six samples were unable to be taken before the chemotherapy dose was given and four post-final dose samples could not be taken due to patient death. Due to chemotherapy being stopped early, 14 patients had a post-final chemotherapy NfL sample taken a median of 22 days (range 10–143) postdose. Figure 3 shows sNfL concentrations after each cycle for the CAPOX (capecitabine and oxaliplatin, n=22) and FOLFOX (folinic acid, 5-fluorouracil and oxaliplatin, n=12) regimens. For CAPOX, there was a statistically significant difference between sNfL concentration at baseline and after three cycles of chemotherapy (usually 390 mg/m^2^ of oxaliplatin; p<0.0001) but not after one or two cycles. For FOLFOX, there was a significant difference from baseline after five cycles of oxaliplatin (usually 425 mg/m^2^; p=0.016) but not after one, two, three or four cycles. After greater numbers of cycles, patient numbers were too small to perform comparisons.

### Relationships between peak sNfL and clinical/oncological factors in platinum-treated patients

**Figure 4:**
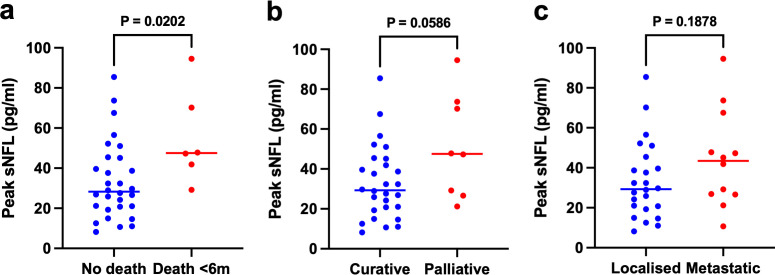
Peak sNfL and oncological variables in patients receiving platinum agents. Median peak sNfL was higher in patients who died within 6 months of study entry (47.5 pg/mL) compared with those who survived (28.3 pg/mL) (**a**). Median peak sNfL was not significantly higher in patients treated with palliative (47.5 pg/mL) compared with curative (29.3 pg/mL) intent (**b**), nor in those with metastatic disease (43.5 pg/mL) compared with localised disease (29.3 pg/mL) (**c**). sNfL, serum neurofilament light chain.

Relationships between unadjusted and z-scored peak sNfL and clinical/neuropathy outcome measures are detailed in table 2. In brief, peak unadjusted sNfL was significantly associated with increasing age (p=0.014), lower BSA (p=0.041) and was higher in patients who died within 6 months of study entry (p=0.020). Five of the six patients who died within 6 months had known metastases at baseline, and the relationship with death was non-significant when the two cisplatin-treated patients were excluded (p=0.063). Peak sNfL was not significantly associated with treatment intent (palliative vs curative; p=0.059) or cancer stage (metastatic vs localised disease; p=0.19). Relationships between peak sNfL and oncological variables in patients who received platinum agents are shown in figure 4. There were no significant associations between peak sNfL and sex, cancer type, length of treatment, total oxaliplatin dose or the presence of diabetes, previous neurotoxic chemotherapy, CKD or excessive alcohol consumption.

**Table 2:** Relationships between peak sNfL (unadjusted and z-scores), clinical characteristics and outcome measures in platinum-treated patients

Continuous comparisons	Unadjusted sNfL		sNfL z-scores	
	Statistic	P value	Statistic	P value
Age	rs=+0.40	0.014	N/A	N/A
BSA	rs=−0.34	0.041	rs=−0.24	0.16
Months of treatment	rs=+0.12	0.47	rs=+0.22	0.20
Total oxaliplatin dose, mg/m^2^ (n=34)	rs=+0.16	0.38	rs=+0.14	0.41
Cumulative dose at last assessment, mg/m^2^ (n=34)	rs=+0.15	0.41	rs=+0.24	0.17
Cancer type	H=6.90 (df=3)	0.075	F=1.12 (df=3)	0.36

Group comparisons	Median sNfL (IQR), pg/mL	P value	Mean sNfL±SD, pg/mL	P value
Sex		0.63		0.47
Male (n=21)	29.22 (21.1–49.4)		2.08±0.99	
Female (n=15)	39.63 (20.8–47.3)		2.30±0.84	
Diabetes mellitus		0.35		0.49
Yes (n=6)	29.5 (18.4–38.7)		2.32±0.44	
No (n=30)	31.0 (21.1–51.3)		2.14±0.99	
Previous neurotoxic chemotherapy		0.45		0.38
Yes (n=4)	23.9 (21.1–40.8)		2.46±0.58	
No (n=32)	32.4 (21.7–50.2)		2.13±0.96	
Alcohol consumption >30 units/week		0.68		0.71
Yes (n=4)	29.1 (27.2–31.6)		2.25±0.34	
No (n=32)	35.0 (20.9–50.2)		2.16±0.97	
Chronic kidney disease		0.11		<0.0001
Yes (n=2)	65.5 (45.5–85.5)		3.14±0.071	
No (n=34)	29.5 (21.0–47.4)		2.11±0.92	
Treatment intent		0.059		0.017
Palliative (n=8)	47.5 (27.3–72.9)		2.66±0.47	
Curative (n=28)	29.3 (19.6–44.3)		2.03±0.97	
Cancer stage		0.19		0.19
Metastatic (n=12)	43.5 (26.7–62.6)		2.43±0.73	
Localised (n=24)	29.3 (19.6–44.0)		2.04±0.99	
Death within 6 months		0.020		0.013
Yes (n=6)	47.5 (38.7–76.3)		2.78±0.47	
No (n=30)	28.3 (20.4–45.2)		2.05±0.95	

Neuropathy outcome measures	Statistic	P value	Statistic	P value
CIPN-RODS	rs=−0.31	0.095	rs=−0.45	0.012
TNSc	rs=+0.30	0.12	rs=+0.37	0.050
TNSc quartile		0.28		0.0007
Grade II–III (n=4)	Median 47.6 (32.0–66.8)		Mean 2.92±0.17	
Grade 0–I (n=25)	Median 32.4 (22.6–49.4)		Mean 2.28±0.69	
Final NCI-CTCAE score		0.11		0.015
Grade 2 (n=3)	Median 52.2 (38.8–70.2)		Mean 2.82±0.28	
Grade 0–1 (n=32)	Median 29.1 (20.9–46.8)		Mean 2.10±0.95	
Neuropathy screening questionnaires all negative		0.016		0.086
Yes (n=6)	Median 16.9 (10.1–31.3)		1.16±1.37	
No (n=30)	Median 35.6 (25.5–51.3)		2.36±0.67	

BSA, body surface area; CIPN-RODS, Chemotherapy-induced Peripheral Neuropathy Rasch-transformed Overall Disability Scale; N/A, not assessed; NCI-CTCAE, National Cancer Institute Common Terminology Criteria for Adverse Events; sNfL, serum neurofilament light chain; TNSc, Total Neuropathy Score-Clinical version.

After conversion to age-adjusted sNfL z-scores, there were significant associations between peak sNfL and CKD (p<0.0001), death within 6 months (p=0.013) and palliative treatment intent (p=0.017). There was no significant association with cancer stage (p=0.19) or any of the other clinical variables.

### Relationships between peak sNfL and neuropathy outcome measures in platinum-treated patients

**Figure 5:**
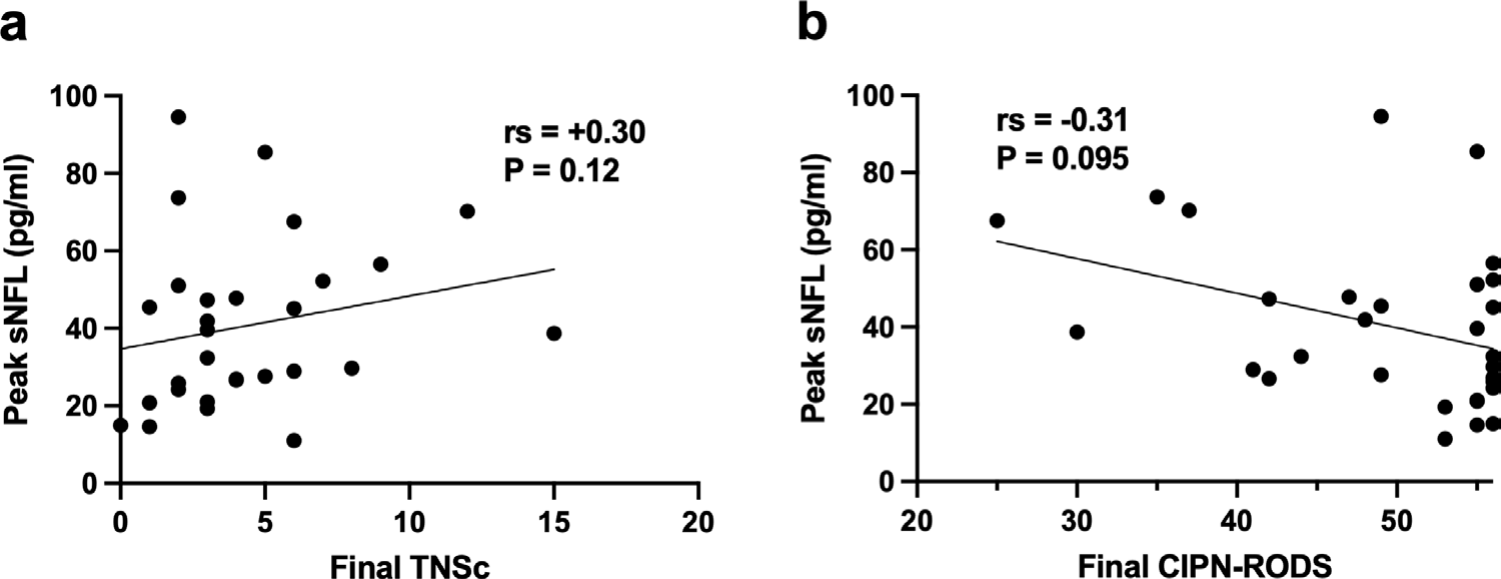
There were no significant correlations between peak sNfL and final TNSc (**a**) or CIPN-RODS (**b**) in patients who received platinum agents. CIPN-RODS, CIPN-Rasch-built Overall Disability Scale; sNfL, serum neurofilament light chain; TNSc, Total Neuropathy Score-Clinical version.

Six patients (17%) had negative screening questionnaires for their entire chemotherapy course and were not included in the CIPN-RODS or TNSc comparisons. Peak unadjusted sNfL was not significantly correlated with final CIPN-RODS (p=0.095) or TNSc (p=0.12) in platinum-treated patients (figure 5). Peak sNfL was not significantly associated with TNSc quartiles (p=0.28) or final CTCAE Grade (p=0.11); however, it was lower in patients with all screening questionnaires negative than in patients who developed neuropathic symptoms (p=0.016).

Peak sNfL z-score showed moderate correlation with lower final CIPN-RODS (rs=−0.45, p=0.012). There was no significant correlation with final TNSc (p=0.050); however, peak sNfL z-score was higher in patients with a final TNSc Grade of II–III (2.92±0.17) compared with patients with a final TNSc Grade of 0–I (2.29±0.69; p=0.0007). Peak sNfL z-score was also higher in patients with a final CTCAE Grade of 2 (2.82±0.28) compared with those with a final Grade of 0–1 (2.10±0.95; p=0.015). There was no significant association between peak sNfL z-score and screening questionnaire results (p=0.086).

### sNfL and outcome measures in taxane-treated patients

Due to low numbers of patients receiving taxanes, peak sNfL was only compared with the continuous outcome measures (TNSc and CIPN-RODS), and there were no significant associations using unadjusted sNfL values or z-scores. One patient receiving taxanes had a final CTCAE-S grade of 2; the other five had a final grade of 0–1.

### Individual patient trends

**Figure 6:**
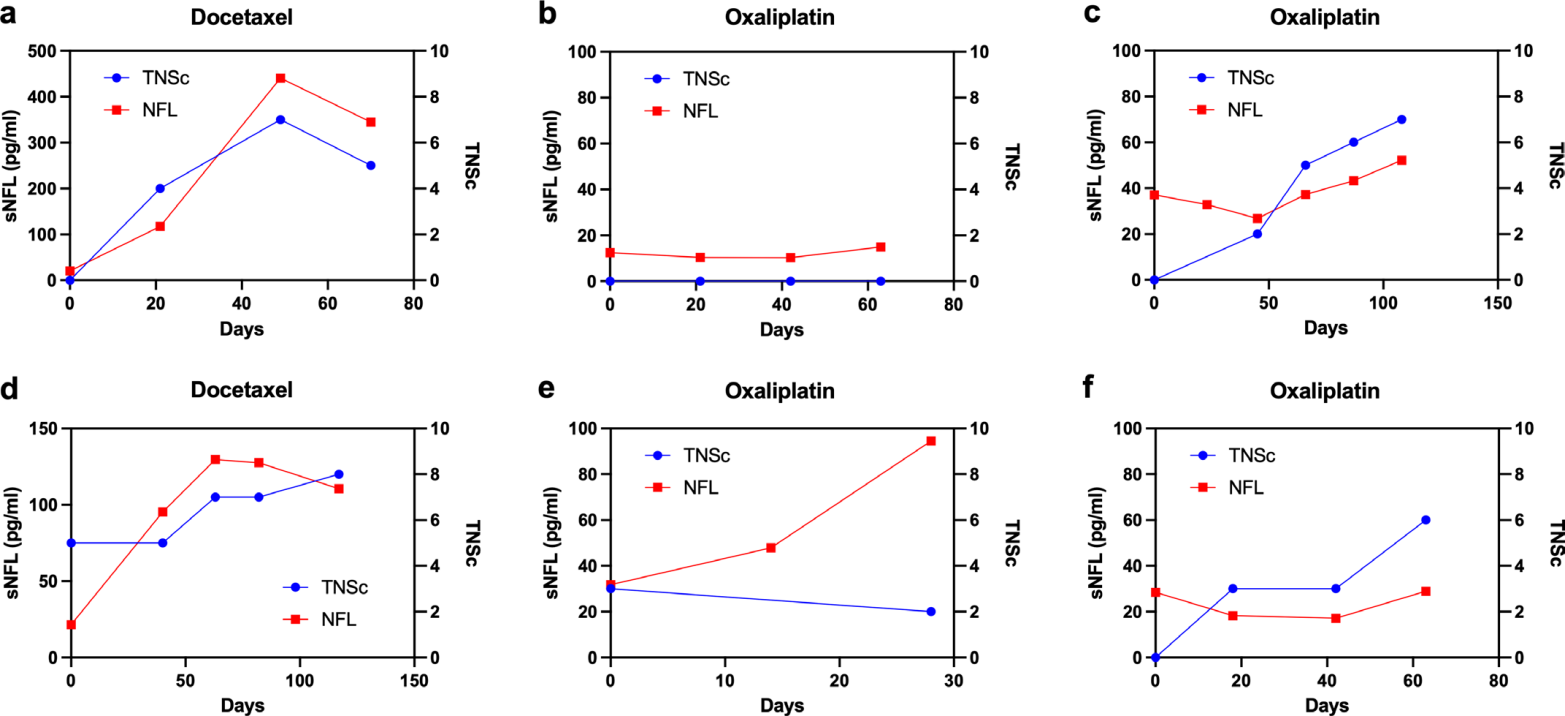
Examples of individual patient trends in sNfL and TNSc. ‘Days’ on the x-axis refers to the number of days since the first dose of chemotherapy. In the majority of patients, increases in sNfL and TNSc occurred concurrently (**a**). In five patients neither sNfL nor TNSc rose during their chemotherapy course, indicating no clinical or biochemical evidence of neuropathy (**b**). In four patients TNSc rose one or two cycles prior to a later increase in sNfL (**c**). In three patients sNfL rose one cycle prior to TNSc (**d**). In three patients there was a rise in sNfL without a significant rise in TNSc, meaning sNfL may have risen for reasons other than neuropathy (**e**). One patient had a rise in TNSc without a concurrent increase in sNfL concentrations (**f**). sNfL, serum neurofilament light chain; TNSc, total neuropathy score clinical version.

In the 33 patients with a TNSc performed after baseline that indicated the development of possible neuropathic symptoms, there were six different trajectory patterns based on the temporal changes of NfL and TNSc:

1. sNfL and TNSc both rise at the same time (17/33, 52%); example shown in figure 6a.
2. No significant rise in either sNfL or TNSc (5/33, 15%); example in figure 6b.
3. TNSc rises one to two cycles before sNfL (4/33, 12%); example in figure 6c.
4. sNfL rises one cycle before TNSc (3/33, 9%); example in figure 6d.
5. Rise in sNfL but no rise in TNSc (3/33, 9%); example in figure 6e.
6. Rise in TNSc but no significant change in sNfL (1/33, 3%); example in figure 6f.

Examining individual elements of the TNSc, 25 of 33 patients (76%) had a rise in their TNSc during the study suggesting the development of CIPN, with 14 of these 25 developing both symptoms and signs and 11 developing examination abnormalities only. Of the 25 patients with a rise in TNSc, 16 developed examination abnormalities (eg, reduced sensation, reduced ankle reflexes) as the first feature of neuropathy before the onset of symptoms, whereas nine had symptoms and signs develop concurrently.

## Discussion

Our study examined the role of serial sNfL measurements in a cohort of patients receiving neurotoxic chemotherapy with close clinical monitoring. For a biomarker to be most useful in clinical practice, it must provide clear enough data in any one individual to guide, support or change therapy.

Consistent with other studies,^8^,^1416 17 25^ sNfL increased in most patients as more cycles of neurotoxic chemotherapy were given, but sNfL was not able to detect early neuronal damage earlier than clinical screening tools in most patients. Since most patients in the study received platinum agents, the analysis was primarily focused on this group. In platinum-treated patients sNfL concentrations were higher with more severe CIPN as measured by TNSc, CIPN-RODS and NCI-CTCAE; however, the differences were relatively modest and only significant when converting raw sNfL values to age-adjusted z-scores. Peak sNfL was generally much higher in patients receiving taxanes and rose more quickly and sharply, suggesting more rapid and severe axonal damage with these agents compared with platinums. Platinum-induced neuropathy is thought to be mediated by the formation of adducts between platinum and DNA, oxidative stress and direct mitochondrial damage, leading to neuronal apoptosis.^26^ In contrast, taxanes cause neuropathy through a variety of mechanisms including inflammation, microtubule damage and mitochondrial changes, leading to axonal degeneration.^27^ As a result of these different mechanisms, it is possible that sNfL is less useful for detecting CIPN caused by platinums compared with taxanes and, similar to our study, previous studies have found sharper and more dramatic increases in sNfL with taxanes^9^,^1214 16^ compared with platinum agents.^8 16 25^ While platinum and taxane-treated groups differed in some respects in our study, their baseline sNfL concentrations were similar, meaning that these differences are unlikely to explain the higher sNfL levels seen with taxane treatment. In another study of sNfL with oxaliplatin, most of the sNfL rise occurred between months 3 and 6 and there were no significant sNfL differences between CTCAE Grades 0–1 and 2, even after 6 months of treatment.^8^ Our analysis found similar results, with modest sNfL rises during 3-month oxaliplatin courses, which in the UK in 2025 are much more common than 6-month courses. Other reports on oxaliplatin have also found modest NfL increases,^16 25^ with one study reporting a weaker correlation between TNSc and NfL with oxaliplatin than with paclitaxel.^16^ The results of our study and others suggest a limited ability of sNfL to predict clinically significant neuropathy with platinum agents.

We found an association between sNfL and death within 6 months of study entry in patients receiving platinum agents, and, using age-adjusted z-scores, it was also higher in patients receiving treatment with palliative intent compared with curative intent. These associations are likely to have been confounded by other factors including cancer stage and severity; however, the trends seen in our study are worthy of further exploration in larger studies, to see whether sNfL associates with non-neuropathy factors in people with cancer.

Individual patient trends showed that increases in sNfL closely mirrored increases in TNSc in the majority of patients, meaning that sNfL and TNSc rose at the same time in most patients developing neuropathy, and in most patients who developed no significant symptoms or signs of neuropathy, the sNfL remained relatively unchanged over the chemotherapy course. However, in only 3 of the 25 patients with clinical evidence of neuropathy (12%; 2 treated with oxaliplatin, 1 with docetaxel) did sNfL rise before any neuropathy could be identified by the TNSc. Thus, for most patients, sNfL does not offer an advantage over careful clinical assessment despite its accuracy in predicting the development of neuropathy in the majority of patients. Given the relatively slow and modest increases in sNfL seen with platinum agents, we feel it is unlikely that the blood sampling frequency was too low to detect early NfL rises prior to the development of clinical signs; however, we cannot exclude this as a possibility.

In the real-world oncology setting, clinicians seldom have time to perform a neurological examination on all patients receiving neurotoxic chemotherapy. Additionally, the neurological examinations in this study were performed by a specialist peripheral nerve neurologist who may have been able to detect early clinical signs of neuropathy more easily than other clinicians. One could thus argue that sNfL could be used in place of a clinical examination or act as an additional marker prior to a clinical appointment; however, it is uncertain whether such an approach would improve clinical outcomes or be cost-effective. Additionally, 9% of patients released significant amounts of sNfL into the circulation without developing clinical evidence of neuropathy. While it is possible that these patients may have developed neuropathy after the study ended due to the ‘coasting’ phenomenon, the rise in sNfL could have been the result of non-neuropathy factors. Using sNfL to determine dose reduction or cessation of chemotherapy might then lead to unnecessary suspension. Similar findings were seen in a study of 190 patients who received carboplatin and paclitaxel for ovarian cancer, where a number of patients had very high sNfL levels after five or six cycles despite being asymptomatic or having only very mild neuropathy.^12^ NfL is not specific for peripheral nerve damage, with a multitude of CNS conditions leading to high NfL levels.^6^ Additionally, most patients in our study had gastrointestinal cancers and in some cases, NfL may have been released due to axonal damage to the enteric nervous system from disease or surgery.

This study has a number of limitations. There were fewer patients recruited than desired, which meant the study may have been underpowered to detect associations between sNfL and the neuropathy outcome measures and some of the oncological variables. Additionally, the use of a screening questionnaire meant that some patients did not contribute to the TNSc and CIPN-RODS analyses. Only three platinum-treated patients developed Grade 2 CIPN, and it is possible that sNfL would have been more strongly associated with neuropathy outcome measures if greater numbers of platinum-treated patients had developed more severe neuropathy. The associations between sNfL and neuropathy outcome measures were only significant when NfL was converted to age-adjusted z-scores, suggesting age may have masked some true associations. This emphasises the importance of considering age when interpreting NfL values. Many patients had dose reductions or had chemotherapy stopped early due to side effects including neuropathy. This could underestimate the neurotoxic effect of platinum agents, as patients receiving longer courses were more likely to be those with less severe neuropathic symptoms. Analysis of individual patient trends relied on visual analysis of trajectory patterns, and more complex analysis of graphical trends could have strengthened this data. We also recruited patients receiving a variety of different chemotherapy drugs and treatment regimens. This meant that we had a mixed sample and were unable to look for trends in NfL and neuropathy based on standardised time-points. We chose a mixed sample because when this research was conceived, NfL had not been studied in human patients with CIPN and we hoped to broadly explore the response of sNfL to a variety of agents. However, we could only recruit small numbers of patients receiving taxanes, mainly because the COVID-19 pandemic meant that many patients were prescribed alternative treatments to chemotherapy to reduce the risk of immunosuppression. Thus, the study results mainly apply to platinum agents.

Our study also has a number of strengths, including the detailed neurological assessment of symptomatic patients with each chemotherapy cycle. This allowed us to examine individual trends and compare the timing of changes in clinical symptoms/signs and sNfL levels. We also examined relationships between sNfL and non-neuropathy factors. These associations are important to understand if NfL is to be considered as a potential biomarker for CIPN in clinical practice, as there may be reasons other than neuropathy causing an NfL rise. Future studies including larger numbers of patients would be important to look further for associations between sNfL and oncological factors.

## Conclusions

This study suggests that taxanes cause more rapid and severe axonal damage than platinum agents. While age-adjusted sNfL z-scores associate with neuropathy severity in platinum-treated patients, careful clinical assessment by a neurologist is likely to detect the onset of neuropathy at a similar time to sNfL. Given the relatively modest increases in sNfL seen with platinum agents in this study, sNfL is less likely to be useful for diagnosing and monitoring platinum-induced CIPN than the CIPN due to taxanes. We hope that ongoing research into peripheral nerve specific biomarkers could lead to a more specific biomarker that could be useful in the oncology setting.

## Supplementary material

10.1136/bmjno-2025-001312 online supplemental table 1

## Data Availability

Data are available upon reasonable request.
