# Supplementary material for: Prospective assessment of serum neurofilament light chain in platinum-induced and taxane-induced peripheral neuropathy
Source: BMJ Neurol Open. 2026 Jan 5;8(1):e001312. doi: 10.1136/bmjno-2025-001312 (PMC12778218; doi:10.1136/bmjno-2025-001312)
Supplement: online supplemental table 1 [file bmjno-8-1-s001.docx]

**Supplementary Table 1**

Modified Michigan Neuropathy Screening Instrument^1^

| **Question (Yes/No)** |
| --- |
| Do you have a numb feeling in your arms, hands, legs and/or feet? |
| Do you ever have any burning pain in your arms, hands, legs and/or feet? |
| Are your arms, hands, legs and/or feet too sensitive to touch? |
| Do you ever have any prickling feelings in your arms, hands, legs and/or feet? |
| Does it hurt when the bed covers touch your skin? |
| When you get into the bath or shower, are you able to tell the hot water from the cold water? |
| Do you have weakness of the muscles in your arms, hands, legs and/or feet? |
| Do your legs hurt when you walk? |
| Are you able to sense your feet when you walk? |
| When you walk, is your balance impaired? |

1. Feldman EL, Stevens MJ, Thomas PK, Brown MB, Canal N, Greene DA. A practical two-step quantitative clinical and electrophysiological assessment for the diagnosis and staging of diabetic neuropathy. Diabetes Care. 1994;17(11):1281-1289.
